# Sprachentwicklungstest für drei- bis fünfjährige Kinder (3;0–5;11 Jahre) – Diagnostik der Sprachentwicklung und der auditiven Gedächtnisleistungen: Retrospektive Auswertung der Ergebnisse bei Kindern mit bilateraler Cochleaimplantatversorgung im Kindesalter

**DOI:** 10.1007/s00106-025-01631-2

**Published:** 2025-04-07

**Authors:** Barbara Streicher, Kerstin Kreibohm-Strauß, Stefanie Kröger, Dominique Kronesser, Yvonne Seebens, Cynthia Glaubitz, Dennis Metzeld, Ruth Lang-Roth

**Affiliations:** 1 Cochlear Implant Centrum (CIK), Uniklinik Köln Hals‑, Nasen‑, Ohrenklinik & Kopf- und Halschirurgie, Kerpener Straße 62, 50931 Köln, Deutschland; 2 Auf der Bult – Zentrum für Kinder und Jugendliche, Cochlear Implant Centrum „Wilhelm Hirte“, Hannover, Deutschland; 3 Klinik und Poliklinik für Hals-Nasen-Ohrenheilkunde, Sektion Implant Centrum (ICF), Universitätsklinikum Freiburg, Medizinische Fakultät, Albert-Ludwigs-Universität Freiburg, Freiburg, Deutschland; 4 Sächsischen CI-Zentrum, Universitätsklinikum Carl Gustav Carus an der Technischen Universität Dresden, Dresden, Deutschland; 5 Hör- und Sprachförderung Rhein – Main GmbH, Cochlear Implant Centrum (CIC) Rhein-Main, Friedberg, Deutschland; 6 Hals-Nasen-Ohrenklinik, Kopf- und Halschirurgie, Cochlear-Implant-Centrum CICERO, Uniklinikum Erlangen, FAU Erlangen-Nürnberg, Erlangen, Deutschland

**Keywords:** Sprachentwicklungsstörungen, Sprachentwicklung, Rehabilitations-Outcome, Prothesen und Implantate, Rehabilitation, Language development disorders, Language development, Rehabilitation outcome, Prostheses and implants, Rehabilitation

## Abstract

**Hintergrund:**

Die Versorgung mit einem Cochleaimplantat (CI) beeinflusst die Ausbildung des auditiven Gedächtnisses und die Sprachentwicklung. Letztere ist eine Voraussetzung für den Erwerb der Schriftsprache und des Lesens und somit entscheidend für die spätere Bildungsbiografie.

**Material und Methoden:**

Die Sprachentwicklungstests für drei- bis fünfjährige Kinder (SETK 3–5) wurden zur Beurteilung der rezeptiven und produktiven Sprachentwicklung sowie der auditiven Gedächtnisleistung während der Folgetherapie eingesetzt. Mit einer retrospektiven Querschnittsstudie wurden die Daten von Kindern, die bilateral mit Cochleaimplantaten (CI) versorgt wurden, aus 6 CI-Zentren der ACIR (Arbeitsgemeinschaft CI Rehabilitation) ausgewertet. Die Unterteilung der Studiengruppe erfolgt in Gruppen, die einerseits anhand des Lebensalters (LA) und anderseits nach ihrer Hörerfahrung mit CI, dem „Höralter“ (HA), ausgewertet wurden. Darüber hinaus wurde differenziert nach CI-Operation vor dem ersten Lebensjahr (CI ≤ 12 Monate) und CI-Operation ab dem zweiten Lebensjahr bis zum vierten Lebensjahr (CI > 12 ≤ 48 Monate). So entstehen die Gruppen für den SETK 3;0–3;11 Jahre: G1LA (CI ≤ 12 Monate), G2LA (CI > 12 ≤ 48 Monate), G1HA (CI ≤ 12 Monate), G2HA (CI > 12 ≤ 48 Monate). Für den SETK 4;0–5;11 Jahre sind es folgende Gruppen: G3LA (CI ≤ 12 Monate); G4LA (CI > 12 ≤ 48 Monate), G3HA (CI ≤ 12 Monate) und G4HA (CI > 12 ≤ 48 Monate).

**Ergebnisse:**

Die Gruppen G1LA, G1HA, G3LA, G3HA (CI ≤ 12 Monate) erreichen altersgerechte Werte in allen Untertests des SETK (3–5). Kinder, die im zweiten Lebensjahr versorgt wurden, holen im Spracherwerb auf. Einige Kinder entwickeln ebenfalls sprachliche Fähigkeiten, jedoch liegen die T-Werte unterhalb der Norm in Bezug zum Lebensalter. Kinder, die mit Deutsch als Zweitsprache aufwachsen, zeigen Entwicklungen im Zweitspracherwerb. Ihre Ergebnisse sind in den Auswertungen bezogen auf das Lebensalter und der Hörerfahrung mit CI nicht altersgerecht.

**Schlussfolgerung:**

Die frühe Versorgung im ersten Lebensjahr ist eine wichtige Voraussetzung dafür, dass Kinder mit CI-Versorgung sprachliche Fähigkeiten entwickeln, die altersgerecht sind. Dennoch ist die Streuung innerhalb der Gruppen breit, sodass Sprachentwicklungsdiagnostik und Therapie im Rahmen der Folgetherapie während des primären Spracherwerbs erforderlich ist, damit Verzögerungen während des primären Spracherwerbs rechtzeitig erkannt werden.

## Abstract (en)

**Background:**

Fitting of a cochlear implant (CI) influences the development of auditory memory and thus the development of speech. Speech development is a prerequisite for the acquisition of written language and reading and, thus, the later educational biography.

**Materials and methods:**

Language development tests for 3–5-year-old children (SETK 3–5) represent a standardized procedure to assess receptive and expressive speech development as well as auditory memory. In a retrospective cross-sectional study, data of children who were fitted with bilateral CIs from six CI centers of the ACIR (*Arbeitsgemeinschaft CI Rehabilitation*) were evaluated. The study group was divided into groups that were analyzed on the basis of age (chronological age; LA) and hearing experience with CI (hearing age; HA). In addition, a distinction was made between CI surgery before the age of 1 year (CI ≤ 12 months) and CI surgery from the second to the fourth year of life (CI > 12 ≤ 48 months). The study group was then subdivided according to the SETK 3;0–3;11 years test into G1LA (CI ≤ 12 months), G2LA (CI > 12 ≤ 48 months), G1HA (CI ≤ 12 months), and G2HA (CI > 12 ≤ 48 months). The study group for the SETK 4;0–5;11 years test was subdivided into G3LA (CI ≤ 12 months), G4LA (CI > 12 ≤ 48 months), G3HA (CI ≤ 12 months), and G4HA (CI > 12 ≤ 48 months).

**Results:**

The groups G1LA, G1HA, G3LA, and G3HA (CI ≤ 12 months) achieved age-appropriate values in all subtests of the SETK (3–5). Children who receive CI within the second year of life catch up in terms of language acquisition and some also develop language skills, but the T scores are below the age-adapted norm values. Children with a multilingual background show results below the norm of their hearing peers in both test settings (chronological age and hearing age).

**Conclusion:**

Early provision of CI in the first year of life is an important prerequisite for children to develop age-appropriate language skills. Nevertheless, there is wide variation within the groups, so that speech diagnostics and therapy are required as part of follow-up treatment during primary language acquisition to detect and avoid major language delay.

## Frühe Sprachentwicklung

Sprachkompetenz bestimmt in hohem Maße das Denken und Handeln, und sie prägt die spätere Bildungsbiografie maßgeblich [15, 29]. Die Ausbildung der gesprochenen Sprache entscheidet über den zukünftigen Erwerb der Schriftsprache und des Lesens [5, 14]. Kinder mit einer Cochleaimplantat(CI)-Versorgung vor dem ersten Lebensjahr haben heute die Möglichkeit, eine altersgerechte Sprachkompetenz mit einem guten phonologischen Bewusstsein zu erlangen [7, 16, 23, 29]. Gleichzeitig wird in Studien beschrieben, dass unterschiedliche, heterogene Verläufe des frühen Spracherwerbs nach CI-Versorgung existieren [20, 27]. Für die Planung, die vorausschauenden Förderung und Therapie ist es deshalb erforderlich, die frühe Sprachentwicklung zu untersuchen [3, 12, 20].

### Spracherwerb bei Kindern mit Cochleaimplantatversorgung

Kinder mit einer kongenital erworbenen Ertaubung und einer unauffälligen allgemeinen Entwicklung können durch die Versorgung mit einem Cochleaimplantat (CI) in die Lage versetzt werden, einen unauffälligen primären Spracherwerb zu durchlaufen. Nach dem neurokognitiven Modell von Kronenberger und Pisoni [20] vollzieht sich der Spracherwerb über 2 Kanäle: einerseits über die schnelle Informationsverarbeitung, in deren Verlauf sich der phonologische Speicher und das Lexikon mit der Bedeutung (Semantik) von Sprache ausbilden, und andererseits mit dem langsameren Weg der komplexen kognitiven Verarbeitung von Sprache.

### Hörbahnreifung

In den ersten 2 Lebensjahren des Kindes kommt deshalb der Hörbahnreifung und der Synaptogenese eine besondere Bedeutung zu, sodass von einer sensiblen Phase für die Ausbildung des Hörens und der Sprache auszugehen ist [19, 32]. Das Kind eignet sich während dieses Prozesses ein eigenes Kenntnissystem an, indem es lernt, unterschiedliche Komponenten zusammen zu setzen: die suprasegmentale, die phonetisch-phonologische, die morphosyntaktische und die kommunikativ-pragmatische Komponente [15].

### Phonologische Schleife

Der Spracherwerb und die Entwicklung des phonologischen Arbeitsgedächtnisses (phonologische Schleife) sind eng miteinander verknüpft, da Gehörtes repräsentiert, identifiziert und durch die Sprachproduktion artikuliert wird [15, 21]. Nur aus gespeicherten sprachlichen Einheiten können die der Sprache zugrunde liegenden suprasegmentalen und segmentalen Bestandteile abgeleitet werden [15, 20]. Hierdurch bildet sich das Wissenssystem aus, das wiederum die Grundlage für das Sprachverstehen und die sprachliche Produktion darstellt. Besonders der Ausbildung des phonologischen Arbeitsgedächtnisses kommt eine zentrale Rolle für die Qualität des Wortschatzaufbaus und der weiteren Ausbildung des Sprachverstehens und der Sprachproduktion zu [10, 15, 32].

Für den Erwerb der rezeptiven und expressiven Sprache hat die bilaterale Versorgung mit CI-Systemen vor dem ersten Lebensalter deshalb eine große Relevanz, damit die Diskrepanz zwischen der Sprachentwicklung auf Basis der Hörerfahrung mit CI und der altersgerechten Entwicklung so gering wie möglich ist [12, 26, 32].

### Meilensteine

Zur Sprachentwicklung bei Kindern mit CI sind zahlreiche Studien vorhanden, die auf der einen Seite die Hör- und Sprachentwicklung der Gruppe der Kinder mit CI untersuchen und auf der anderen Seite die Ergebnisse in einen Vergleich mit hörgesunden Kindern stellen.

Innerhalb der Gruppe der Kinder mit CI werden vielfach heterogene Verläufe aufgezeigt [6, 12, 17, 33]. Übersichtsarbeiten zeigen ebenfalls, dass viele früh versorgte Kinder die Meilensteine nicht erreichen, sondern bis zu 2 Standardabweichungen von der zu erwartenden Sprachentwicklung mit CI abweichen [17, 27]. Die Meilensteine umfassen den Wortschatzaufbau und erste Wortkombinationen, den Auf- und Ausbau der Grammatik, die Entwicklung der Laute auf phonetisch-phonologischer Ebene sowie den Gebrauch von Sprache in sozial-pragmatischen Kontexten [2, 28]. Teilweise kommunizieren Kinder lautsprachlich noch nicht entsprechend ihrem Lebensalter, oder sie nutzen Gebärdensysteme [8, 12]. Andererseits ist eine Annäherung an die Meilensteine der Sprachentwicklung von normalhörenden Kindern zu beobachten [11]. Weitere Untersuchungen deuten darauf hin, dass dies umso schneller geschieht, je früher die Kinder implantatversorgt werden [8, 12, 18, 23, 24].

Die Entwicklung von Kindern mit CI, die nach der Erstanpassung des Sprachprozessors sehr schnell die frühen Meilensteine der Sprachentwicklung (erste Wörter oder Wortkombinationen) von hörenden Kindern erreichen und zu den hörenden Altersgenossen aufschließen, werden genauso beschrieben wie Kinder mit einer verlangsamten Sprachentwicklung. Man spricht hier von „gap-closer“ und „gap-opener“ [16]. In einer weiteren Untersuchung [33], die 52 Datensätze des SETK 3–5, auswertet hatte, zeigen Kinder, die vor dem ersten Lebensjahr CI-versorgt werden, altersgerechte Werte für die rezeptive und produktive Sprache. Der Verlauf der Sprachentwicklung bei Kindern, die nach dem ersten Lebensjahr versorgt werden, kann ebenfalls überprüft werden, jedoch kann der SETK 3–5 nicht entsprechend dem Lebensalter ausgewertet werden, sodass Unterschiede von bis zu einer Standardabweichung zur altersgerechten Sprachentwicklung bestehen [33, 34].

In einer Übersichtsarbeit von Ruben [27], die Veröffentlichungen zu der Entwicklung der rezeptiven und expressiven Sprache untersucht, zeigen die Ergebnisse aus 17 Arbeiten an insgesamt 904 Probanden, dass sowohl auf der Ebene des Sprachverstehens (rezeptive Sprache), als auch auf der Ebene der Sprachproduktion (expressive Sprache) weniger als 80 % der Kinder mit CI Normwerte erreichen. In 8 weiteren Studien wird die frühe CI-Versorgung um 12 Monate mit CI als positiver Effekt für die Sprachentwicklung beschrieben [23]. Je später die Versorgung durchgeführt wird, desto schlechter ist die Prognose für die weitere Ausbildung der expressiven und rezeptiven Sprache.

Inzwischen postuliert die Leitlinie „Therapie von Sprachentwicklungsstörungen“, dass die Überprüfung der Sprachentwicklung bei Kindern mit Hörschädigung mit an hörenden Kindern normierten Verfahren geschehen sollte und dass die Auswertung am Lebensalter gemessen werden soll [9]. Die Fachkraft ordnet die individuelle Entwicklung inklusive der Hörerfahrung mit CI entsprechend in die allgemeine Entwicklung ein.

### Faktoren für den normalen Spracherwerb

Die Literatur nennt unterschiedliche Faktoren für den normalen Spracherwerb nach CI-Versorgung. In vielen Fällen sind deutliche Aufholtendenzen während der Sprachentwicklung zu beobachten, in anderen Fällen stagniert die Entwicklung oder bleibt sogar hinter den Erwartungen zurück. Neben den Einflussfaktoren der Nutzungsdauer und Trageakzeptanz des CI-Systems [4, 13, 25] und des elterlichen Sprachinputs werden folgende den Spracherwerb beeinflussende Faktoren beschrieben [4, 27, 32]:

- Audiologische Faktoren: Früherkennung und Versorgung der Hörschädigung, frühe Implantation, bilaterale CI-Versorgung oder bimodale Versorgung, intaktes neurologisches System, bestehende Resthörigkeit
- Kindbezogene Faktoren: altersgemäße nonverbale kognitive Fähigkeiten, altersentsprechende sprachauditive Merkfähigkeit, keine Zusatzbeeinträchtigungen
- Faktoren des kindlichen Umfelds: Bildungsniveau der Eltern, sozioökonomischer Status der Eltern, Qualität der Eltern-Kind-Interaktion, Familiengröße, Mehrsprachigkeit

In einer großen multizentrischen Studie wurde erstmals die Sprachentwicklung von Kindern mit kongenitaler Ertaubung nach bilateraler CI-Versorgung bis zum Alter von 48 Monaten untersucht. Die Annahme ist, dass Kinder mit CI-Versorgung sich im Verlauf dem Spracherwerbsprofil hörender Kinder annähern. Folgende Hypothesen sollen untersucht werden:

1. Die CI-Versorgung vor dem ersten Lebensjahr (≤ 12 Monate) beeinflusst den Spracherwerb so, dass sich die sprachlichen Fähigkeiten denen von gut hörenden Kindern annähert.
2. Das auditive Arbeitsgedächtnis (phonologisches Arbeitsgedächtnis und Satzgedächtnis) bildet sich bei Kindern, die im Alter von unter einem Jahr versorgt werden, altersgerecht aus.

## Material und Methoden

Die vorliegende retrospektive Datenanalyse erfolgte im Rahmen eines umfangreich angelegten multizentrischen Projekts der Zentren der Arbeitsgemeinschaft CI Rehabilitation e. V. (ACIR).

### Test- und Fragebogenverfahren zur Hör- und Sprachentwicklung

Der Sprachentwicklungstest für drei- bis fünfjährige Kinder (SETK 3–5) ist für den Altersbereich 3;0 bis 5;11 Jahre ein standardisiertes Verfahren zur Beurteilung der rezeptiven und produktiven Sprachentwicklung und des phonologischen Gedächtnisses bei hörenden Kindern [15, 30].

Zunächst gedacht als Testverfahren, das frühzeitig die Sprachverzögerung bei hörenden Kindern erkennt, kommt dieses Testverfahren zur Verlaufskontrolle nach CI-Versorgung in vielen etablierten CI-Zentren zum Einsatz [13, 33, 34]. Als behandlungsbedürftig gelten Kinder, deren T‑Werte unter 40 (Normwert 40–60) liegen. Die Aufgaben sind so konstruiert, dass sie geeignet sind, die Kompetenz der Kinder zum Verstehen und zur Bildung von sprachlichen Strukturen sowie ihre sprachlichen Verarbeitungsfähigkeiten (phonologisches Arbeitsgedächtnis) zu beurteilen.

Das Testmaterial besteht aus Bildkarten für das Materialset wie Verstehen von Sätzen (VS), Enkodierung semantischer Strukturen (ESR), morphologische Regelbildung (MR) und einem Figurensatz Phonologisches Arbeitsgedächtnis (PGN). Der Protokollbogen für 3;0–3;11 Jahre kann in 2 Altersgruppen (I 3;0–3;11 Jahre/II 4;0–4;11 Jahre) ausgewertet werden. Die Version für die Altersgruppe 4;0–5;11 beinhaltet die Untertests: Verstehen von Sätzen (VS), Morphologische Regelbildung (MR), Phonologisches Arbeitsgedächtnis (PGN) und Satzgedächtnis (SG) mit der Auswertung nach Altersgruppen (III 4;0–4;5 Jahre, IV4;6–4;11 Jahre, V 5;0–5;5 Jahre, VI 5;6–5;11 Jahre). Die Untertests phonologisches Arbeitsgedächtnis und Satzgedächtnis untersuchen das Sprachgedächtnis des Probanden. Die Untergliederung des Tests in 2 altersspezifische Teile beruht auf der Annahme, dass zwischen dem dritten und vierten Lebensjahr ein qualitativer Sprung der Sprachentwicklung geschieht. In der Regel beträgt die Testdauer 20–30 min. Der SETK 3–5 bildet ebenfalls den Erwerbsverlauf der Zweitsprache ab und kann somit bei Kindern zum Einsatz kommen, die Deutsch als Zweitsprache erwerben [15].

Insgesamt wurden 315 Tests in der Altersstufe 3;0 bis 3;11 Jahre und 308 in der Altersstufe 4;0 bis 5;11 Jahre durchgeführt. Die Daten mussten nachbearbeitet werden, weil meist das „Höralter“ für die Auswertung herangezogen wurde. So wurden die Rohwerte (Tab. 1) in die entsprechenden T‑Werte der Altersgruppen (I, II, III, IV, V, VI) überführt.

**Tab. 1:** Bearbeitung und Auswahl des Datensatzes

Testverfahren	SETK 3;0–3;11 Jahre				SETK 4;0–5;11 Jahre			
Datensatz insgesamt aus 6 Zentren der ACIR	*n* = 315 → *n* = 283 erfüllen die Einschlusskriterien				*n* = 308 → *n* = 269 erfüllen die Einschlusskriterien			
Gruppierung und Auswertung der T‑Werte nach Altersgruppen	Lebensalter 3;0–3;11 Jahre (I/II)		„Höralter“ 3;0–3;11 Jahre (I/II)		Lebensalter 4;0–5;11 Jahre (III/IV/V/VI)		„Höralter“ 4;0–5;11 Jahre (III/IV/V/VI)	
Anzahl der ausgewerteten Datensätze	*n* = 167		*n* = 116		*n* = 192		*n* = 77	
Gruppierung der Untergruppen/Altersangaben in Monaten	*G1LA*	*G2LA*	*G1HA*	*G2HA*	*G3LA*	*G4LA*	*G3HA*	*G4HA*
	*CI * *≤* *12*	*CI * *>* *12* *≤* *48*	*CI * *≤* *12*	*CI* *>* *12* *≤* *48*	*CI* *≤* *12*	*CI* *>* *12* *≤* *48*	*CI* *≤* *12*	*CI* *>* *12* *≤* *48*
	*n* *=* *83*	*n* *=* *84*	*n* *=* *61*	*n* *=* *55*	*n* *=* *95*	*n* *=* *97*	*n* *=* *49*	*n* *=* *28*

Die Sprachentwicklungstests SETK 3;0–3;11 und SETK 4;0–5;11 Jahre unterscheiden sich in 2 Untertests. Die Rohwerte der Untertests des SETK 3;0–3;11 Jahre wurde entsprechend der Altersgruppe I und II ausgewertet. Der Sprachentwicklungstest SETK 4;0 bis 5;11 Jahre wurde entsprechend der Altersgruppe III, IV, V und VI ausgewertet. Die Gruppenbildung erfolgte nach dem Kriterium des Zeitpunkts der Versorgung mit dem ersten CI (CI ≤ 12 Monate/CI > 12 ≤ 48 Monate) und der Auswertung der Testergebnisse nach Lebensalter (LA) und „Höralter“ (HA). G1LA, G2LA, G3LA und G4LA wurden nach dem Lebensalter ausgewertet. G1HA, G2HA, G3HA, G4HA zeigen Auswertungen, die nach dem „Höralter“ vorgenommen wurden

*ACIR *Arbeitsgemeinschaft CI Rehabilitation,* CI *Cochleaimplantat,* HA* „Höralter“, *LA *Lebensalter, *SETK* Sprachentwicklungstest für drei- bis fünfjährige Kinder (3;0–5;11 Jahre)

### Bearbeitung und Auswertung des Sprachentwicklungstests (SETK 3–5)

Der Datensatz umfasst Ergebnisse des SETK 3–5 aus 6 CI-Zentren, die zwischen 2003 und 2020 erhoben wurden. Aufgrund der unterschiedlichen Altersbereiche zum Zeitpunkt der Implantation wurde folgende Aufteilung für den SETK 3;0–3;11 Jahre vorgenommen (Tab. 1): G1LA (CI ≤ 12 Monate, *n* = 83), G2LA (CI > 12 ≤ 48 Monate, *n* = 84), G1HA (CI ≤ 12 Monate, *n* = 62), G2HA (CI > 12 ≤ 48 Monate, *n* = 55).

Für den SETK 4;0–5;11 Jahre erfolgte diese Einteilung: G3LA (CI ≤ 12 Monate, *n* = 95); G4LA (CI > 12 ≤ 48, *n* = 97), G3HA (CI ≤ 12 Monate, *n* = 49) und G4HA (CI > 12 ≤ 48 Monate, *n* = 28). Ausgeschlossen wurden Daten von Kindern, die nicht mit den Einschlusskriterien (kongenital bilateral ertaubt, bilateral versorgt innerhalb von 12 Monaten, Versorgung bis zum 48 Lebensmonat, keine bekannten zusätzlichen Behinderungen zum Zeitpunkt der Überprüfung) vereinbar waren.

### Beschreibung der Stichprobe

Die Stichprobe umfasst, wie in Tab. 2 und 3 dargelegt, demnach 283 Kinder für den SETK 3;0 bis 3;11 und 269 Kinder für den SETK 4;0 bis 5;11 Jahre. Die G1LA und G2LA, deren T‑Werte bezogen auf das Lebensalter ausgewertet werden, umfassen insgesamt 167 (♀ = 85, ♂ = 82) Daten. Die G1HA und G2HA, deren T‑Werte bezogen auf das „Höralter“ mit CI ausgewertet wurde, beinhalten 116 (♀ = 61, ♂ = 55) Kinder. Es konnten 192 Probanden (♀93, ♂ = 99) in G3LA und G4LA ausgewertet werden. Die Gruppen G3HA und G4HA bestehen aus 77 (♀40, ♂ = 37) Kindern.

**Tab. 2:** Beschreibung der Stichprobe 1 insgesamt und die Aufteilung in die Untergruppen SETK 3;0 bis 3;11 Jahre sowie SETK 4;0 bis 5;11 Jahre

–		*Sprachentwicklungstest für Kinder (SETK 3;0–5;11 Jahre)*							
		SETK 3;0–3;11 Jahre				SETK 4;0–5;11 Jahre			
Gruppe	Gesamt	Stichprobe LA (G1/G2)	Anteil (%)	Stichprobe HA (G1/G2)	Anteil (%)	Stichprobe LA (G3/G4)	Anteil (%)	Stichprobe HA (G3/G4)	Anteil (%)
Anzahl *n*		167	–	116	–	192	–	77	–
*Geschlecht*									
m		85	50,9	61	52,5	93	48,4	40	51,9
w		82	49,1	55	47,5	99	51,6	37	48,1
*Ätiologie*									
Unbekannt		81	48,5	15	12,8	67	34,9	12	15,6
Genetisch		33	19,8	30	25,9	60	31,3	26	33,8
Infektion		14	8,4	11	9,5	13	6,8	4	5,2
Andere		39	23,4	51	44	52	27,1	35	45,5
k. A.		–		9	7,8	–			
*Sprache*									
Deutsch		140	81,4	80	68,9	155	80,7	57	74
Andere Lautsprache		14	8,1	16	13,8	21	10,9	7	9,1
Gebärdensprache		6	3,5	5	4,4	5	2,6	1	1,3
k. A.		14	7	15	12,9	11	12	12	15,6
*CI-Herstellerfirma*									
Cochlear		83	49,7	48	41,3	116	53	40	51,9
ME-DEL		35	21,0	36	31,1	36	16,4	19	24,7
Advanced Bionics		24	14,4	4	3,4	32	14,6	3	3,9
k. A.		25	15	28	24,1	35	16	15	19,5
*Op.-Differenz ≤* *6 Monate*	*n* = 325	–							
*Op.-Differenz >* *6 Monate*	*n* = 56								

*CI *Cochleaimplantat,* HA* „Höralter“, *k. A.* keine Angaben,* LA *Lebensalter

**Tab. 3:** Beschreibung der Stichprobe 2 in Bezug auf das Lebensalter (LA) und „Höralter“ (HA) bei Versorgung der rechten und linken Seite, der Erstanpassung rechts und links, des Lebens- und Höralters bei Testung und der Tragedauer der Cochleaimplantat(CI)-Systeme

Angaben in Monaten	*Sprachentwicklungstest für Kinder (SETK 3;0–5;11 Jahre)*											
	SETK 3;0–3;11 Jahre						SETK 4;0–5;11 Jahre					
*Lebensalter bei CI rechts*	*Gesamt LA*	*G1LA *	*G2LA *	*Gesamt HA*	*G1HA *	*G2HA *	*Gesamt LA*	*G3LA *	*G4LA *	*Gesamt HA*	*G3HA *	*G4HA *
*n*	*167*	*83*	*84*	*116*	*61*	*55*	*192*	*95*	*97*	*77*	*49*	*28*
Min	5,8	5,8	9,7	4,7	4,7	10,7	4,7	4,7	11,3	4,7	4,7	11,2
Max	33,3	22	33,3	48,6	17,7	45,9	47,2	17,7	47,2	46,3	16,3	46,3
Mittelwert	13,2	8,8	17,4	14,7	9,1	20,5	14,7	9,1	20,3	12,5	8,9	18,5
Median	11,2	8,4	16,3	11,4	8,6	17,6	11,9	8,9	17,5	10,4	8,7	14,2
SD	6,2	2,3	5,9	9,3	2,4	10,7	8,2	2,2	8,2	7,4	2,2	9,1
*Lebensalter bei CI links*												
Min	5,8	5,8	11	6	6	10	4,8	4,8	11,2	4,8	4,8	11,2
Max	38,4	19,6	38,4	48,6	19,6	45,9	49,3	20,5	49,3	46,3	20,5	46,3
Mittelwert	13,9	9,5	18,2	15,9	10,1	19,9	15,4	9,7	21	13,7	10,2	19,6
Median	11,7	9,1	17,5	13,2	9,1	21,7	12,7	9,1	18,8	11,8	9,4	17,6
SD	6,4	2,5	6,3	9,2	3,1	8,9	8,4	2,9	8,4	7,4	3,2	8,7
*Lebensalter bei Erstanpassung CI rechts*												
Min	6,9	6,9	12,2	5,7	5,8	12,1	5,8	5,8	12,7	5,8	5,8	12,8
Max	41,5	22,9	41,5	49,9	18,6	47	48,3	18,6	48,3	47,3	17,8	47,3
Mittelwert	14,5	10	18,8	15,9	10,2	21,7	15,9	10,3	21,5	13,7	10,2	19,7
Median	12,5	9,6	17,6	12,5	9,6	18,7	13,2	10,3	19	11,6	10,1	15,3
SD	6,4	2,2	6,2	9,4	2,4	9,6	8,2	2,1	8,1	7,2	2,2	8,9
*Lebensalter bei Erstanpassung CI links*												
Min	6,9	6,9	12,2	7,1	7	12,1	5,8	5,8	12,3	5,8	5,8	12,8
Max	41,5	21,5	41,5	49,9	21,5	47	56,8	22,2	56,4	47,3	22,2	47,3
Mittelwert	15,5	10,7	19,6	17,1	11,3	22,9	16,9	12,2	22,5	14,9	11,5	20,9
Median	12,9	10,4	18,4	14,3	10,5	21,2	13,9	11,1	20	13	10,7	19,1
SD	6,6	2,5	6,5	9,3	3,1	8,9	8,8	2,9	9	7,4	3,3	8,7
*Lebensalter bei Testung*												
Min	36,6	37,1	36,6	44,4	44,4	49,3	49	49,3	49	54,5	54,5	62,9
Max	48	48	48	86,6	58,4	85	70,7	69,7	70,7	97,3	76,3	97,3
Mittelwert	43,2	43,5	43	54,8	48,9	60,8	59	58,4	59,5	67,2	63,3	73,9
Median	44,2	44,6	44,1	52,2	47,9	59	58,7	58,4	59,4	65,3	62,8	72,9
SD	3,4	3,2	3,6	9,6	3,9	9,4	5,8	5,4	6,2	8,1	4,4	8,5
*Tragedauer mit CI seit Erstanpassung*												
Min	8,3	26,2	8,3	36	36,1	36	12,9	38,3	12,9	26,8	49	49,1
Max	40	40	35,6	48	48	47,9	60,3	60,3	56,9	47,5	64,7	62,9
Mittelwert	29,4	34	24,9	39,7	39,5	40	43,5	48,7	38,5	37,7	53,8	54,6
Median	30,6	34,7	24,9	38,2	38	38,9	45,5	48,8	39,5	53,9	53,6	54,7
SD	6,9	3,4	6,5	3,4	3,5	3,4	9,6	5,4	10,9	5,2	3,9	4,4

*G1LA *(CI ≤ 12 Monate), *G2LA* (CI > 12 ≤ 48 Monate), *G1HA* (CI ≤ 12 Monate), *G2HA* (CI > 12 ≤ 48 Monate) *G3LA* (CI ≤ 12 Monate), *G4LA* (CI > 12 ≤ 48 Monate), *G3HA* (CI ≤ 12 Monate), *G4HA* (CI > 12 ≤ 48 Monate), *Min *Minimum,* Max *Maximum,* SD* Standardabweichung, *SETK* Sprachentwicklungstest für drei- bis fünfjährige Kinder (3;0–5;11 Jahre)

Bekannte Ursachen der Hörschädigung und die Daten zu den Herstellern sind ebenfalls in Tab. 2 aufgeführt. In die Auswertung wurden Kinder mit CI, die Deutsch als Zweitsprache erwerben, eingebunden. Insgesamt waren es 30 Kinder mit CI in der Altersgruppe 3;0 bis 3;11 Jahre und 28 in der Altersgruppe 4;0 bis 5;11 Jahre.

Mit Gebärdensprache wurden 6 Kinder in der Altersgruppe 3;0–3;11 Jahre und 9 Kinder in die Altersgruppe 4;0–5;11 Jahre eingebunden. Ob es sich um den Gebärdeneinsatz im Sinne der die Lautsprache unterstützenden Gebärde oder um die deutsche Gebärdensprache handelte und die Kinder einen additiven Lautspracherwerb durchliefen, konnte auf Basis der Dateneingabe nicht nachvollzogen werden. Innerhalb der ersten 6 Monate wurden 325 Kinder bilateral mit CI-Systemen versorgt.

### Datenauswertung und Statistik

Die statistische Auswertung der hier dargestellten demografischen Daten erfolgte durch die Studienleitung in Freiburg und das beteiligte Zentrum in Köln. Zur Auswertung wurden die Programme IBM SPSS Statistics 29 (SPSS Inc., Chicago, IL, USA) und Microsoft Excel 2019 (Microsoft, Redwood, WA, USA) verwendet. Zur Ergebnisdarstellung wurden deskriptive statistische Verfahren gewählt. Die abgebildeten Boxplots zeigen Median und Perzentile. Der Interquartilsabstand (Box) definiert das 25. und das 75. Perzentil, die Fehlerbalken das 10. bzw. 90. Perzentil, Ausreißer liegen jeweils darunter bzw. darüber. Die Überprüfung der Normalverteilung erfolgte mit dem Kolmogorov-Smirnov(KS)-Test (nach Andrei Nikolajewitsch Kolmogorov und Nikolai Wassilijewitsch Smirnov). Da die Verteilung die Normalverteilungsvoraussetzung verletzt, die Verteilungsform zwischen den Gruppen nach Kolmogorov-Smirnov aber hinreichend gleich ist, wurde der Mann-Whitney-U-Test eingesetzt. Zusätzlich wurde die Normalverteilung mit und ohne die mehrsprachigen Kinder geprüft. In beiden Fällen sind die Daten nicht normalverteilt, weder nach Kolmogorov-Smirnov noch nach Shapiro-Wilk. Die Effektstärke wurde auf Basis der Z‑Werte mit der Pearson-Korrelation berechnet. Das Signifikanzniveau wurde auf *p* < 0,05 festgelegt.

## Ergebnisse

### Auswertung des Sprachentwicklungstests für Kinder SETK 3;0–3;11 Jahre

Kinder der Gruppe G1LA (CI ≤ 12 Monate), deren mittleres Lebensalter zum Testzeitpunkt 43,5 (SD ± 3,2) Monate und deren CI-Tragedauer 34 (SD ± 3,4) Monate betrug, zeigten ein relativ homogenes Profil mit T‑Werten im mittleren Normbereich und somit altersgerechte sprachliche Fähigkeiten. Kinder der Gruppe G2LA (CI > 12 ≤ 48 Monate), deren mittleres Lebensalter zum Testzeitpunkt 43 (SD ± 3,6) Monate und deren CI-Tragedauer 24,9 Monate (SD ± 6,5) betrug, hatten in den Untertests Verstehen von Sätzen, Enkodierung semantischer Strukturen und Phonologisches Arbeitsgedächtnis Mediane unter 40 (T-Wert). Im Untertest Morphologische Regelbildung lag der Median für den T‑Wert bei 40.

Im Vergleich zeigte die Gruppe G1HA (CI ≤ 12 Monate) mit einem Lebensalter von 48,9 (SD ± 3,9) Monaten und einer CI-Tragedauer von 39,5 (SD ± 3,5) Monaten altersgerechte T‑Werte.

Gruppe G2HA (CI > 12 ≤ 48 Monate) war mit einem Lebensalter von 60,8 (SD ± 9,4) Monaten und einer Tragedauer von 40 (SD ± 3,4) Monaten ebenfalls knapp altersgerecht in 4 Untertests.

**Abb. 1:**
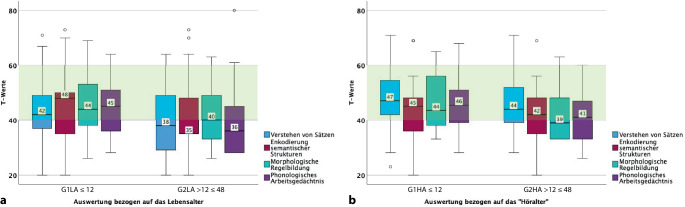
Ergebnisse des SETK 3;0–3; 11 Jahre, nach dem Lebensalter (*LA*) und nach dem „Höralter“ (*HA*) ausgewertet. In den *Boxplots *Untertests Verstehen von Sätzen, Enkodierung semantischer Strukturen, Morphologische Regelbildung und Phonologisches Arbeitsgedächtnis dargestellt. In der Auswertung Unterscheidung der Altersgruppen *G1LA* und *G1HA* (CI ≤ 12 Monate) sowie *G2LA* und *G2HA* (CI > 12 ≤ 48 Monate). *y‑Achse* T‑Werte. *Hellgrün hinterlegter Bereich* T‑Werte zwischen 40 und 60, im Normbereich

Die Ergebnisse der Gruppen streuten in den Untertests (Verstehen von Sätzen, Enkodierung semantischer Strukturen, Phonologisches Arbeitsgedächtnis, Morphologische Regelbildung; Abb. 1).

### Auswertung des Sprachentwicklungstests für Kinder SETK 4;0–5;11 Jahre

Gruppe G3LA (CI ≤ 12 Monate), deren mittleres Lebensalter zum Testzeitpunkt 58,4 (SD ± 5,4) Monate und deren CI-Tragedauer 48,7 (SD ± 5,4) Monate betrug, zeigte ein homogenes Profil mit T‑Werten im unteren bis mittleren Normbereich und somit altersgerechte sprachliche Fähigkeiten. Die Kinder der Gruppe G4LA (CI > 12 ≤ 48 Monate), die zum Testzeitpunkt 59,5 (SD ± 6,2) Monate alt waren und deren CI-Tragedauer 38,5 (SD ± 10,9) Monate betrug, erreichten in allen Untertests T‑Werte, die nicht altersgerecht waren.

Im Vergleich zeigte Gruppe G3HA (CI ≤ 12 Monate) mit 63,2 (SD ± 4,5) Monaten und einer CI-Tragedauer von 63,8 (SD ± 4,4) Monaten altersgerechte T‑Werte.

**Abb. 2:**
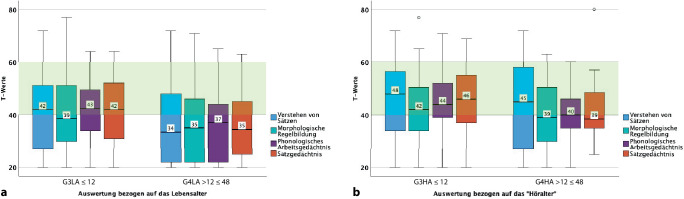
Ergebnisse des SETK 4;0–5; 11 Jahre, nach dem Lebensalter (*LA*) und nach dem „Höralter“ (*HA*) ausgewertet. In den *Boxplots* Untertests Verstehen von Sätzen, Morphologische Regelbildung, Phonologisches Arbeitsgedächtnis und Satzgedächtnis dargestellt. In der Auswertung Unterscheidung der Altersgruppen *G3CI* und *G3HA* (CI ≤ 12 Monate) sowie *G4LA* und *G4HA* (CI > 12 ≤ 48 Monate). *y‑Achse* T‑Werte. *Hellgrün hinterlegter Bereich* T‑Werte zwischen 40 und 60, im Normbereich

Die Gruppe G4HA (CI > 12 ≤ 48 Monate) zeigte mit einem Lebensalter von 73,9 (SD ± 8,5) Monaten und einer CI-Tragedauer von 54,6 (SD ± 4,4) Monaten ebenfalls Werte in der unteren Norm in sämtlichen Untertests. Insgesamt betrachtet ist Gruppe G4HA (CI > 12 ≤ 48 Monate) etwa 2 Monate älter als das vorgesehene Alter der hörgesunden Normgruppe, das laut Handbuch bei 71 Monaten liegt (Abb. 2).

### Auswertung des Sprachentwicklungstests für Kinder SETK 3;0–3;11 und 4;0–5;11 Jahre bei Kindern, die mehrsprachig in einer anderen Lautsprache aufwachsen

**Abb. 3:**
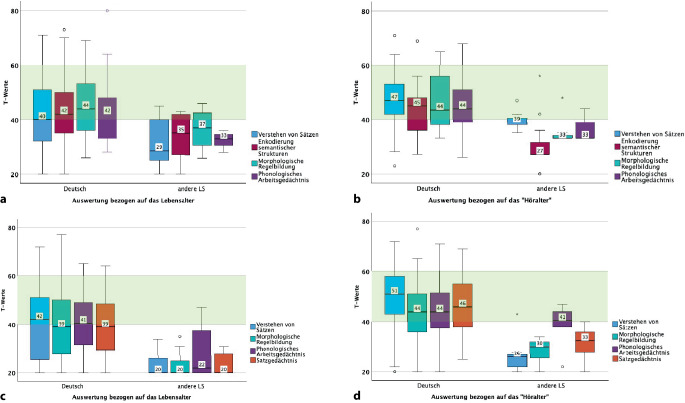
**a,b** Ergebnisse der Subgruppe der Kinder, die in einer anderen Lautsprache (*LS*) aufwachsen, im Unterschied zu den Kindern mit Cochleaimplantat (CI), die monolingual Deutsch aufwachsen. Ergebnisse des SETK 3;0–3;11 Jahre, differenziert nach **a** Lebensalter (*LA*) und **b** „Höralter“ (*HA*) dargestellt. *y‑Achse* T‑Werte. *Hellgrün hinterlegter Bereich* T‑Werte zwischen 40 und 60, im Normbereich.** c,d** Ergebnisse der Subgruppe der Kinder, die in einer anderen Lautsprache (*LS*) aufwachsen, im Unterschied zu den Kindern, die monolingual Deutsch aufwachsen. Ergebnisse des SETK 4;0–5;11 Jahre, differenziert nach **c** Lebensalter (*LA*) und **d** „Höralter“ (*HA*) dargestellt. *y‑Achse* T‑Werte. *Hellgrün hinterlegter Bereich* T‑Werte zwischen 40 und 60, im Normbereich

In die Auswertung der Ergebnisse wurden bewusst Kinder mit CI einbezogen, die Deutsch als zweite Sprache erlernen. Mit genügend deutschen Sprachkenntnissen kann der Test orientierend durchgeführt werden. Mit dem SETK 3;0–3;11 Jahre wurden bei insgesamt bei *n* = 30 Kindern mit CI die sprachlichen Fähigkeiten bestimmt. In Abb. 3a,b sind die Ergebnisse dieser Untergruppe in der Auswertung für das Lebensalter und das „Höralter“ dargestellt. Die Subgruppe „andere Lautsprache“ zeigte unabhängig von der Auswertung zum Lebensalter oder „Höralter“ keine altersgerechten T‑Werte. Mit dem SETK 4;0–4;11 Jahre wurden *n* = 28 Kinder überprüft. In Abb. 3c,d sind die Gruppenunterschiede dargestellt. Auch hier erreichte die Gruppe der mehrsprachig aufwachsenden Kinder keine Sprachentwicklungsdaten, die lebens- oder höraltersgerecht waren. Eine Ausnahme stellte der Untertest „Phonologisches Arbeitsgedächtnis“ dar, der zur Auswertung „Höralter“ den T‑Wert von 41 ergab.

### Signifikanzen der Untertests in Abhängigkeit vom Zeitpunkt der Versorgung

Mit dem Mann-Whitney-U-Test wurde verwendet, um den Unterschied in den Rängen zwischen dem Zeitpunkt der Versorgung mit CI zu berechnen.

Die Tab. 4 zeigt die Berechnung der Rangunterschiede zwischen den Altersgruppen mit CI-Versorgung im Alter unter einem Jahr, CI ≤ 12 Monate, und CI-Versorgung nach dem ersten Lebensjahr, CI > 12 ≤ 48 Monate.

**Tab. 4:** Rangunterschiede zwischen dem Zeitpunkt der CI-Versorgung im Alter unter einem Jahr (CI ≤ 12 Monate) und der CI-Versorgung nach dem ersten Lebensjahr (CI > 12 ≤ 48 Monate) und Rangunterschiede der Gruppe „andere Sprache“

*Mann-Whitney-U-Test – asymptotische Signifikanz (2-seitig)*						
Untertests		Verstehen von Sätzen	Enkodierung semantischer Strukturen	Morphologische Regelbildung	Phonologisches Arbeitsgedächtnis	Satzgedächtnis
*SETK 3;0–3;11 Jahre*						
Auswertung zum Lebensalter		U = 1.862.500	U = 2.003.000	U = 1275,00	U = 868,00	Erst in den Altersgruppen III, IV, V, VI
		Z = −1,685	Z = −2,834	Z = −2,272	Z = −2,513	
		*p* = 0,092	*p* = 0,005^**^	*p* = 0,023^*^	*p* = 0,012^*^	
		r = 0,14	r = 0,20	r = 0,20	r = 0,25	
Auswertung zum „Höralter“		U = 450.000	U = 784.500	U = 695.000	U = 535.000	
		Z = −0,835	Z = −1,62	Z = −1,623	Z = −2,113	
		*p* = 0,404	*p* = 0,292	*p* = 0,105	*p* = 0,035^*^	
		r = 0,10	r = 0,11	0,17	r = 0,24	
Auswertung zum Lebensalter	Subgruppe: andere Lautsprache	U = 186.000	U = 210.000	U = 113.500	U = 55.500	
		Z = −1,902	Z = −1,875	Z = −1,502	Z = −1,695	
		*p* = 0,057	*p* = 0,061	*p* = 0,133	*p* = 0,09	
		r = 0,17	r = 0,16	r = 0,14	r = 0,17	
Auswertung zum „Höralter“	Subgruppe: andere Lautsprache	U = 72.000	U = 109.500	U = 111.500	U = 114.500	
		Z = −2,472	Z = −3,916	Z = −3,862	Z = −3,068	
		*p* = 0,013^*^	*p* < 0,001^***^	*p* < 0,001^***^	*p* = 0,002^**^	
		r = 0,30	r = 0,44	r = 0, 44	r = 0,37	
*SETK 4;0–4;11 Jahre*						
Auswertung zum Lebensalter		U = 3.145.500	Nicht in den Altersgruppen III, I, V, V und VI	U = 3.566.000	U = 2.387.000	U = 2.765.500
		Z = −1,800		Z = −2,182	Z = −3,100	Z = −2,961
		*p* = 0,072		*p* = 0,029^*^	*p* = 0,002^**^	*p* = 0,003^**^
		r = 0,13		r = 0,15	r = 0,24	r = 0,22
Auswertung zum „Höralter“		U = 479.500		U = 571.000	U = 356.500	U = 432.500
		Z = −0,533		Z = −0,714	Z = −1,360	Z = −1,487
		*p* = 0,594		*p* = 0,475	*p* = 0,174	*p* = 0,139
		r = 0,06		r = 0,08	r = 0,17	r = 0,17
Auswertung zum Lebensalter	Subgruppe: andere Lautsprache	U = 383.500		U = 413.000	U = 458.000	U = 225.000
		Z = −4,805		Z = −5,086	Z = −3,507	Z = −4,793
		*p* < 0,001^***^		*p* < 0,001^***^	*p* < 0,001^***^	*p* < 0,001^***^
		r = 0,38		r = 0,38	r = 0,28	r = 0,38
Auswertung zum „Höralter“	Subgruppe: andere Lautsprache	U = 40.500		U = 61.000	U = 115.500	U = 45.000
		Z = −3,177		Z = −2,929	Z = −0,977	Z = −2,853
		*p* < 0,001^***^		*p* = 0,003^*^	*p* = 0,329	*p* = 0,004^**^
		r = 0,43		r = 0,37	r = 0,13	r = 0,37

^*^ signifikant, ^**^ sehr signifikant, ^***^ hochsignifikant*, CI *Cochleaimplantat,* SETK* Sprachentwicklungstest für drei- bis fünfjährige Kinder (3;0–5;11 Jahre)

#### Rangunterschiede im Sprachentwicklungstest 3;0–3;11 Jahre

Die mittleren Ränge der Gruppe G1LA und G2LA unterscheiden sich sehr signifikant im Untertest Enkodierung semantischer Strukturen (U = 2.003.000, Z = −2,834, *p* = 0,005, r = 0,20) und signifikant in den Untertests Morphologische Regelbildung (U = 1275,00, Z = 2,272, *p* = 0,023, r = 0,20) und Phonologisches Arbeitsgedächtnis (U = 868,00, Z = 2,513, *p* = 0,012, r = 0,25). In den Gruppen G1HA und G2HA ist die Rangverteilung im Untertest Phonologisches Arbeitsgedächtnis (U = 535.000, Z = 2,113, *p* = 0,035, r = 0,25) signifikant.

#### Rangunterschiede im Sprachentwicklungstest 4;0–5;11 Jahre

Die mittleren Ränge der Gruppe G3LA und G4LA unterscheiden sich sehr signifikant im Untertest Phonologisches Arbeitsgedächtnis (U = 2.387.000, Z = −3,100, *p* = 0,002, r = 0,24) und Satzgedächtnis (U = 2.765.500, Z = −2,961, *p* = 0,003, r = 0,22). Signifikant unterscheiden sich die Gruppen G3LA und G4LA im Untertest Morphologische Regelbildung (U = 3.566.000, Z = −2,182, *p* = 0,029, r = 0,15). In der Gruppe G3HA und G4HA unterscheiden sich die Ränge nicht signifikant.

#### Rangunterschiede in der Untergruppe „andere Lautsprache“

In den Untertests des Sprachentwicklungstest 3;0–3;11 Jahre, ausgewertet in Bezug auf das Lebensalter, bestehen keine Rangunterschiede in der Untergruppe „andere Lautsprache“. Hingegen unterscheidet sich die Rangverteilung im Sprachentwicklungstest 4;0–4;11 Jahre. Hochsignifikant unterscheiden sich die Ränge zur Auswertung in Bezug auf das Lebensalter im Untertest Verstehen von Sätzen (U = 383.500, Z = −4,805, *p* < 0,001; r = 0,38), Morphologische Regelbildung (U = 423.000, Z = −5,086, *p* < 0,001, r = 0,38), Phonologisches Arbeitsgedächtnis (U = 458.000, Z = −3,507, *p* < 0,001, r = 0,28) und Satzgedächtnis (U = 225.000, Z = −4,793, *p* < 0,001, r = 0,38). Auch die Auswertung bezogen auf das „Höralter“ ergibt einen hochsignifikanten Unterschied im Untertest Verstehen von Sätzen (U = 40.500, Z = −3,177, *p* < 0,001, r = 0,43), einen sehr signifikanten Unterschied im Untertest Satzgedächtnis (U = 45.000, Z = −2,853, *p* < 0,004, r = 0,37) und einen signifikanten Unterschied im Untertest Morphologische Regelbildung (U = 61.000, Z = −3,177, *p* = 0,003, r = 0,43). In allen aufgeführten Fällen sind die sprachlichen Fähigkeiten von früh implantatversorgten Kindern und monolingual Deutsch aufwachsenden Kindern besser.

### Prozentuale Verteilung der T-Werte im SETK 3:0–3;11 Jahre und 4;0–5;11 Jahre

**Abb. 4:**
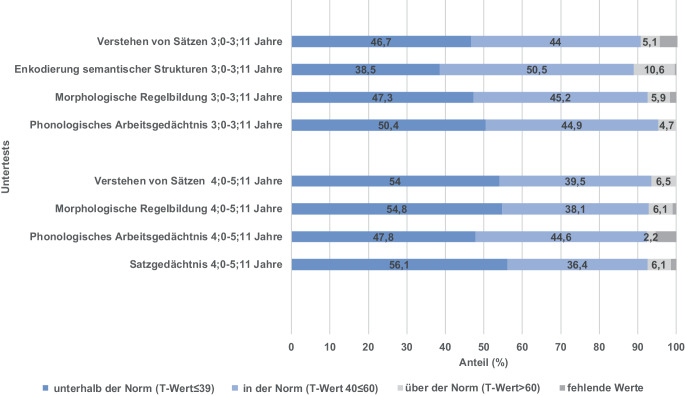
Prozentuale Verteilung der T‑Werte der gesamten Stichprobe. *y‑Achse* Untertests von SETK (3;0–3;11 Jahre, *n* = 283, und 4;0–5;11 Jahre, *n* = 269) aufgeführt. *x‑Achse* Anteil (%). *Dunkelblauer Bereich der Balken* Anteil der T‑Werte ≤ 39, unterhalb der Norm. *Hellblauer Bereich der Balken* Anteil, der T‑Werte zwischen 40 und 60, in der Norm. *Hellgrauer Bereich der Balken* Anteil der T‑Werte über 60, über der Norm. *Dunkelgrauer Bereich der Balken* fehlende Werte des jeweiligen Untertests

In Abb. 4 ist die prozentuale Verteilung der T‑Werte der gesamten Stichprobe aufgeführt. Der SETK 3;0–3;11 Jahre besteht aus 283 Datensätzen und der SETK 4;0–5;11 Jahre aus 269. Nicht alle Kinder haben durchgängig sämtliche Untertests durchgeführt. Deshalb sind die verbliebenen Anteile ausgegraut. Ein T‑Wert zwischen 40 und 60 gilt als altersgerecht.

#### Prozentuale Verteilung im SETK 3;0–3;11 Jahre

Im Untertest Verstehen von Sätzen erreichen 44 % der Kinder altersgerechte T‑Werte, 50,4 % liegen unterhalb der Norm und 4,7 % oberhalb der Norm. Im Untertest Enkodierung semantischer Strukturen sind 50,5 % der Kinder altersgerecht, 38,5 % unterhalb von T‑Wert 40, und 10,6 % erreichen T‑Werte über 60. Im Untertest Morphologische Regelbildung befinden sich 45,2 % der Kinder in der Norm, 47,3 % unterhalb der Norm und 5,9 % über der Norm. Im Untertest Phonologisches Arbeitsgedächtnis sind 44,9 % der Kinder altersgerecht, 50,4 % nicht altersgerecht und 4;7 % oberhalb der Norm von T‑Wert 60.

#### Prozentuale Verteilung im SETK 4;0–5;11 Jahre

Im Untertest Verstehen von Sätzen sind 39,5 % der Kinder altersgerecht, 54 % nicht altersgerecht und 6,5 % über der Norm. Der Untertest Morphologische Regelbildung zeigt 38,1 % der Kinder im Bereich der Altersnorm, 54,8 % unterhalb der Norm und 6,1 % über der Norm. Im Untertest Phonologisches Arbeitsgedächtnis erreichen 44,6 % der Kinder altersgerechte T‑Werte, 47,8 % keine altersgerechten Werte und 2,2 % bessere T‑Werte. Im Untertest Satzgedächtnis sind 39,5 % der Kinder innerhalb der altersgerechten Werte, 54 % unterhalb und 6,5 % darüber.

## Diskussion

### Sprachentwicklung

Diese retrospektive multizentrische Studie der Arbeitsgemeinschaft CI-Rehabilitation (ACIR e. V.) konnte einen Datensatz aus den Erhebungen der Sprachentwicklung generieren, der erstmalig überregionale Daten einer großen Kohorte zur Sprachentwicklung CI-versorgter Kinder auswertet. Die leitende Frage war, ob die frühe CI-Versorgung ein Prädiktor für eine folgende normale Sprachentwicklung ist. Der Vergleich wurde mit Testverfahren überprüft, die an hörenden Kindern normiert wurden. Die Leitfrage war, ob sich die Sprachentwicklung in dieser frühen Phase angleicht. Im Zusammenhang mit der Hörbahnreifung steht dabei die Ausbildung des auditiven Gedächtnisses, das in den Untertests Phonologisches Arbeitsgedächtnis und Satzgedächtnis überprüft wurde.

### Auswertung der Variable Zeitpunkt der Versorgung

Die Ergebnisse der Sprachentwicklungstests (SETK 3–5) demonstrieren heterogene Ergebnisse zwischen den Altersgruppen. Die Überprüfung mit dem SETK 3;0–3;11 Jahre in der Gruppe der Kinder mit CI, die bilateral unter einem Jahr versorgt wurden (CI ≤ 12 Monate), ergab durchschnittliche sprachliche Fähigkeiten im Alter von 43,5 (± 3,2) Monaten. Die CI-Tragedauer betrug 34 (± 3,4) Monate. Auch die Gruppe der Kinder, die mit einem „Höralter“ von 39,5 (± 3,5) Monaten und einem Lebensalter von 48,9 (± 3,9) Monaten getestet wurden, zeigte noch eine altersgerechte Sprachentwicklung. Ebenfalls erreicht die Gruppe G3LA (CI ≤ 12 Monate) im SETK 4;0–5;11 Jahre im Lebensalter von 58,4 (± 5,4) und dem „Höralter“ von 48,7 (± 5,4) Monaten in den Untertests altersgerechte Werte. Dieser erfreuliche Verlauf der Kinder, die im ersten Lebensjahr CI-versorgt wurden, bestätigt den Zusammenhang mit der frühen Hörbahnreifung und Synaptogenese mittels der CI [26, 29, 32]. In dieser Untersuchungsgruppe wurden die Grundlagen für das Erreichen der wesentlichen Meilensteine der Sprachentwicklung geschaffen [21].

Die Diskrepanz zwischen Lebensalter und „Höralter“ vergrößert sich in der Gruppe der Kinder mit CI, die zwischen dem zweiten Lebensjahr und dem vierten Lebensjahr (CI > 12 ≤ 48 Monate) mit einem CI versorgt wurden. Das Lebensalter bei Testung für diese Gruppe lag bei der Überprüfung der Sprachentwicklung mit dem SETK 3;0–3;11 Jahre bei 60,8 (± 9,4) Monaten. Das „Höralter“ betrug 40 (± 3,4) Monate. Zum späteren Überprüfungszeitpunkt mit dem SETK 4;0–4;11 Jahre zeigte die Gruppen G4HA (CI > 12 ≤ 48 Monate) in den Untertests keine altersgerechte Sprachentwicklung bezogen auf das Lebensalter von 58,4 (± 5,4) Monaten und grenzwertige T‑Werte bezogen auf das „Höralter“ von 73,9 (± 8,5) Monaten. Grundsätzlich erreichten Kinder mit CI, die später implantatversorgt wurden, ähnliche Werte, jedoch waren sie zum Zeitpunkt der Überprüfung der Sprachentwicklung älter, sodass die Altersnorm der hörenden Normgruppe nicht oder knapp zutrifft. Han et al. [16] beobachteten in ihren Auswertungen „gap-closer“ und „gap-opener“. In dieser Kohorte waren ähnliche Aufholtendenzen zwischen dem vierten und fünften Lebensjahr zu beobachten [16].

In Kapitel 3 der Leitlinien für Sprachtherapie wird die Auswertung nach dem tatsächlichen Lebensalter der Kinder mit CI als Standard für die Sprachentwicklungsdiagnostik postuliert. Oft galt während der CI-Rehabilitation in der Vergangenheit jedoch das „Höralter“ als Zeitmaß für die Einordnung der sprachlichen Fähigkeiten. Diese Auswertung veranschaulicht, dass das tatsächliche Lebensalter des Kindes bei der CI-Versorgung bis zum ersten Lebensjahr für die Einordnung der Sprachentwicklung zugrunde gelegt werden soll. Zwischen dem dritten und vierten Lebensjahr vollziehen Kinder einen qualitativen Sprung in ihrer Sprachentwicklung. Da die Gruppe der Kinder G1HA und G3HA (CI ≤ 12 Monate) T‑Werte im Normbereich erreicht, scheint mit zunehmender Hörerfahrung mit CI-Systemen dieser Effekt ebenfalls einzutreten.

Eine individuelle Einordung der Sprachentwicklung vor dem Hintergrund der Hörerfahrung mit den CI-Systemen wird notwendig, damit eine gezielte Sprachförderung eingeleitet wird [9]. Jedoch beeinflusst der Zeitpunkt der Versorgung und Dauer der Hörerfahrung mit dem CI wesentlich das Tempo des frühen Spracherwerbs [35]. Die Streuung der Ergebnisse zeigt, dass in der Gruppe der früh bis zum ersten Lebensjahr versorgten Kinder das Spracherwerbsprofil innerhalb der Norm des Lebensalters liegen kann [11, 16, 24, 27]. Andererseits verläuft bei einigen Kindern die Entwicklung langsam oder verzögert. Dies unterstützt die Notwendigkeit der engmaschigen postoperativen Verlaufsdiagnostik im Rahmen der Folgetherapie [1].

### Phonologisches Arbeits- und Satzgedächtnis

Die frühe Möglichkeit der auditorischen Reifung hat Konsequenzen für die Ausbildung des auditiven Gedächtnisses [26, 32]. Dieses wird in den Untertests Phonologisches Arbeitsgedächtnis und Satzgedächtnis überprüft. Die Kinder, die CI-Systeme im ersten Lebensjahr erhielten, zeigten in der Auswertung altersgerechte T‑Werte. Die Rangwerte des Untertests Phonologisches Arbeitsgedächtnis waren signifikant (Tab. 4). Die Streuung innerhalb der Gruppe deutet an, dass einige Kinder dennoch langfristig Probleme in der Gedächtnisleistung hatten. Diese Nachteile manifestierten sich in der Gruppe der Kinder, die nach dem ersten Lebensjahr versorgt wurden. Der signifikante Unterschied zwischen den Gruppen (Tab. 4) unterstreicht, dass die auditive Gedächtnisspanne unterschiedlich ausgeprägt ist [12, 17, 20, 32].

### Mehrsprachigkeit

In dem Datensatz enthalten sind ebenfalls Kinder, die einen bilingualen Spracherwerb durchlaufen. Die Testergebnisse dieser Kinder wurden bewusst in die Studiengruppe eingebunden, da sie Kenntnisse in Deutsch aufwiesen. Die Beobachtung des Verlaufs der Sprachentwicklung ist mittels der Testverfahren gegeben [9]. Der Vergleich der Untertests zeigt bei Kindern, die mehrsprachig aufwachsen, einen signifikanten Unterschied zu der Gruppe der einsprachig aufwachsenden Kinder (Abb. 3a, b). Der Untertest Phonologisches Arbeitsgedächtnis (Abb. 3b) korreliert mit grammatischen Fähigkeiten und dem Sprachverstehen. Damit bildet der Untertest Phonologisches Arbeitsgedächtnis den Verlauf des Spracherwerbs in der zweiten Sprache Deutsch ab [15]. Während der Folgetherapie im Rahmen der CI-Rehabilitation sind insbesondere in der Gruppe der mehrsprachig aufwachsenden Kinder mit CI individuelle Entwicklungsverläufe möglich, und sie sollten entsprechend mit den erforderlichen therapeutischen Maßnahmen unterstützt werden. Jedoch existieren bisher keine validen Normen für Kinder mit einem bilingualen Mehrspracherwerb.

## Bewertung

### Bisherige Untersuchung

Die Versorgung mit einem CI vor dem ersten Lebensjahr wirkt sich positiv auf den primären Spracherwerb und die Ausbildung des auditiven Gedächtnisses aus. Das konnte in dieser ersten multizentrischen Studie für CI-versorgte Kinder in Deutschland nachgewiesen werden. Obwohl keine Normen für Kinder mit Hörschädigung in dem Testverfahren existieren, konnte bei insgesamt 552 Kindern das Testverfahren SETK (3–5) eingesetzt und durchgeführt werden. Im Vergleich mit den Normdaten hörender Kinder konnten ebenfalls die sprachlichen Fähigkeiten von Kindern mit CI-Versorgung beschrieben werden. Insbesondere die Gruppe der Kinder, die vor dem ersten Lebensjahr bilateral mit CI-Systemen versorgt wurden, konnten zum Lebensalter ähnlich wie gesunde Kinder im gleichen Lebensalter getestet und ausgewertet werden. Die Mediane zeigen eine altersgerechte Entwicklung für die Gruppen. Die Streuung innerhalb der Untertests weisen jedoch auch darauf hin, dass der Spracherwerb nicht bei allen Kindern altersgerecht verläuft. Kinder, die einen bilingualen Mehrspracherwerb durchlaufen, konnten ebenfalls mit dem Testverfahren untersucht werden. Aus den Ergebnissen insbesondere im Untertest Phonologisches Arbeitsgedächtnis können Rückschlüsse auf den Beginn des Zweitspracherwerbs gezogen werden.

### Limitationen

Eine etablierte frühkindliche Entwicklungsdiagnostik der allgemeinen und kognitiven Entwicklung war kein Einschlusskriterium. Im Verlauf der kindlichen Entwicklung sind jedoch Verzögerung der allgemeinen und kognitiven Entwicklung möglich, die sich erst im Verlauf ausprägen. Eine allgemeine Verzögerung kann in den ersten Jahren diagnostiziert werden und sich auf die spätere Sprachentwicklung auswirken [22]. Die Studiengruppe stellt eine Auswahl an Daten aus 6 CI-Zentren der Arbeitsgemeinschaft CI Rehabilitation dar. Eine valide Zahl darüber, wie viele der Kinder im Verlauf eine zusätzliche, vielleicht auch leichte Entwicklungsverzögerung haben, fließt in diese Untersuchung nicht ein. Auch wurde nicht systematisch der Input an Sprache innerhalb der Familien erfasst, obwohl dies als positiver Faktor für die zukünftige Sprachentwicklung von Kindern mit Hörschädigung gilt [3, 8, 20, 31]. Kritisch anzumerken ist, dass die Untertests Phonologisches Arbeitsgedächtnis und Satzgedächtnis zwar das auditive Gedächtnis beurteilen, jedoch im weiteren Entwicklungsverlauf durch weitere Testverfahren zum Kurzzeitgedächtnis und zur Merkfähigkeit von suprasegmentalen und segmentalen Strukturen ergänzt werden sollten [5, 10, 14]. Für diese Auswertung wurde der Datensatz auf Basis der Eingangskriterien (bilaterale CI-Versorgung innerhalb eines Jahres, keine erkennbaren Zusatzbehinderungen) bereinigt. Nicht bekannt ist, wie viele Kinder während der Folgetherapie nicht getestet werden konnten, da Mitarbeit oder die Sprachentwicklung dies nicht zuließen.

## Ausblick

Die allgemeine Entwicklungsdiagnostik und Sprachentwicklungsdiagnostik sollte ein fester Bestandteil in der Folgetherapie nach CI-Versorgung sein und durch qualifiziertes Fachpersonal durchgeführt werden, das die individuellen Verläufe diagnostizieren, einordnen und ggf. therapieren kann. Damit zukünftig ein umfassendes Bild über die Sprachentwicklung von CI-Kindern entstehen kann, sollten sämtliche Daten unter Berücksichtigung der linguistischen Ebenen prospektiv erfasst werden.

## Data Availability

Die Daten wurden unter Federführung der Universitätsklinik Freiburg aus sechs Zentren zusammengetragen und stehen anonymisiert und pseudonymisiert zur Verfügung.
